# The Detroit Keloid Scale: A Validated Tool for Rating Keloids

**DOI:** 10.1089/fpsam.2021.0359

**Published:** 2023-03-03

**Authors:** Alexis B. Lyons, David M. Ozog, Henry W. Lim, Kate Viola, Amy Tang, Lamont R. Jones

**Affiliations:** ^1^ Department of Dermatology, Henry Ford Hospital, Detroit, Michigan, USA.; ^2^ Department of Public Health, Henry Ford Hospital, Detroit, Michigan, USA.; ^3^ Department of Otolaryngology Head and Neck Surgery, Henry Ford Hospital, Detroit, Michigan, USA.

## Abstract

**Background:**

Comparing keloid treatment modalities and assessing response to treatments may be predicted by a better classification system.

**Objectives:**

To develop and validate the Detroit Keloid Scale (DKS), a standardized method of keloid assessment.

**Methods:**

Forty-seven physicians were polled to develop the DKS. The scale was validated in 52 patients against the Vancouver Scar Scale (VSS), Patient and Observer Scar Assessment Scale (POSAS), and Dermatology Life Quality Index (DLQI).

**Results:**

The inter-rater reliability was “substantial” for observer DKS and only “moderate” for VSS and observer POSAS (intraclass correlation coefficient were 0.80, 0.60, and 0.47, respectively). Pearson's correlation indicated “moderate” association between observer DKS with observer POSAS (*ρ* = 0.56, *p* < 0.001) and “substantial” relationship between observer DKS and VSS (*ρ* = 0.63, *p* < 0.001). Pearson's correlation indicated “moderate” association between patient portion of DKS and patient portion of POSAS and patient portion of the DKS and DLQI (0.61 and 0.60, respectively, *p* < 0.05). DKS total score consistently showed significant “substantial” relationship with POSAS total score (*ρ* = 0.65, *p* < 0.001).

**Conclusions:**

The DKS offers a validated keloid-specific outcome measure for comparing keloid treatments.

**KEY POINTS**

**Question:** What are the features of keloid scars that affect patients and their treatments?

**Findings:** A new scale was created and compared with existing outcome measures with good success.

**Meaning:** This new scale, the Detroit Keloid Scale, may allow providers to better compare patients with keloids, and the effect of their treatments, leading to research that can improve outcomes.

## Introduction

There is large heterogeneity in the literature when it comes to outcome measures for scars.^1,2^ Outcome measures for scars can include subjective components and objective measurements.^3^ In fact, a recent systematic review identified a total of 40 disease outcome measures that were used in 41 randomized controlled trials.^1^ These range from pliability, firmness, color, thickness, vascularity, surface area, and height among others.^2^ This heterogeneity makes it difficult to compare outcomes of clinical trials and response to treatments, particularly for keloids, which were not included when the most popular scar outcome instruments were designed.

Objective measurements (e.g., ultrasound) of various clinical parameters have been explored in the past to bring objectivity to scar rating systems; however, they often examined hypertrophic scars and did not include those with keloids.^4^ Keloids differ from hypertrophic scars in that they are characterized by horizontal growth beyond the boundary of the original wound and represent a complex pathomechanistic and treatment conundrum.^5^ Moreover, because they disproportionately affect people with skin of color (SOC), common outcome scar scales that often include components such as vascularity are difficult to apply broadly to keloids.

The most commonly used scar outcome measures are the Vancouver Scar Scale (VSS)^6^ and the Patient and Observer Scar Assessment Scale (POSAS).^7^ The VSS, which was developed to rate burn scars, analyzes vascularity, thickness, pliability, and pigmentation by an observer, whereas the POSAS incorporates these attributes plus surface area assessed by an observer as well as pain, itching, color, stiffness, thickness, and relief assessed by the patient.

Although the POSAS and VSS have been utilized to evaluate keloids,^8^ these were not designed specifically with keloids in mind. The Dermatology Life Quality Index (DLQI)^9^ is one of the most consistently utilized patient-reported outcome measures and quantifies how dermatologic skin conditions affect patient quality of life; however, it was not proposed specifically for keloid scars. The objective of this study was to develop and validate a keloid-specific outcome measure, the Detroit Keloid Scale (DKS), to better enable clinicians and researchers to compare keloid treatment modalities among studies and to assess treatment efficacy.

## Materials and Methods

### Development of the DKS

For development of the DKS, physicians from numerous specialties attending the third International Keloid Symposium (Beijing, China, April 19–21, 2019)^10^ and from Henry Ford Hospital (Detroit, MI) were polled through an online survey with item reduction to rank each component in order from most important to least important for the development of the DKS to determine the most important clinical factors when evaluating keloids.

**Fig. 1.**
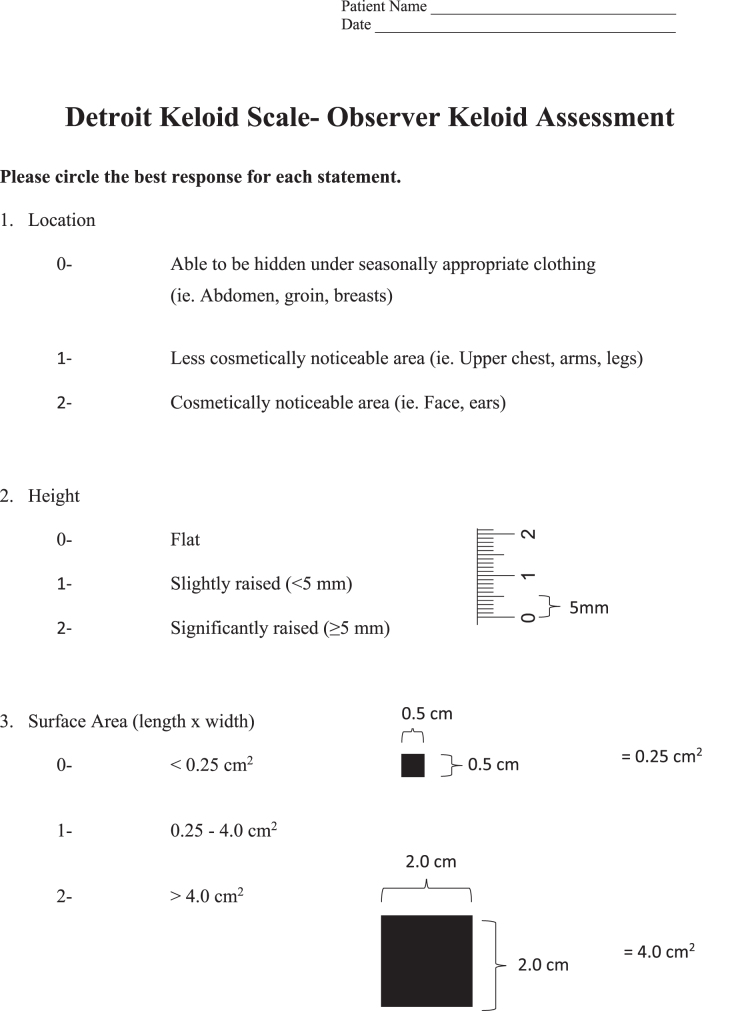

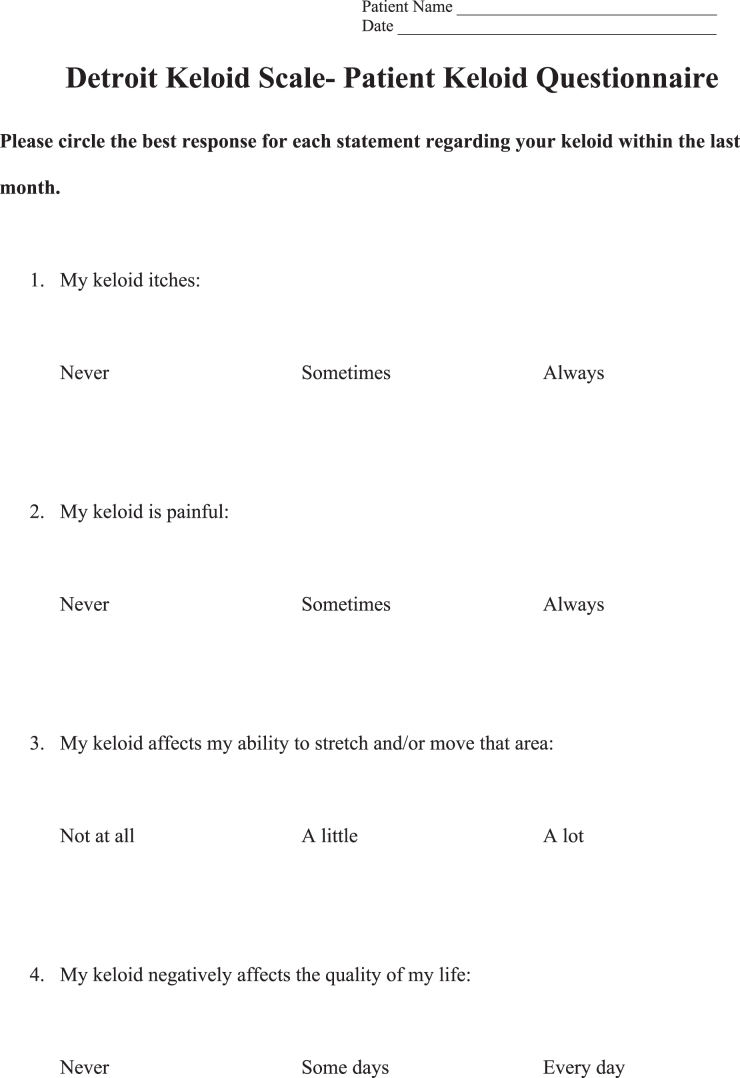

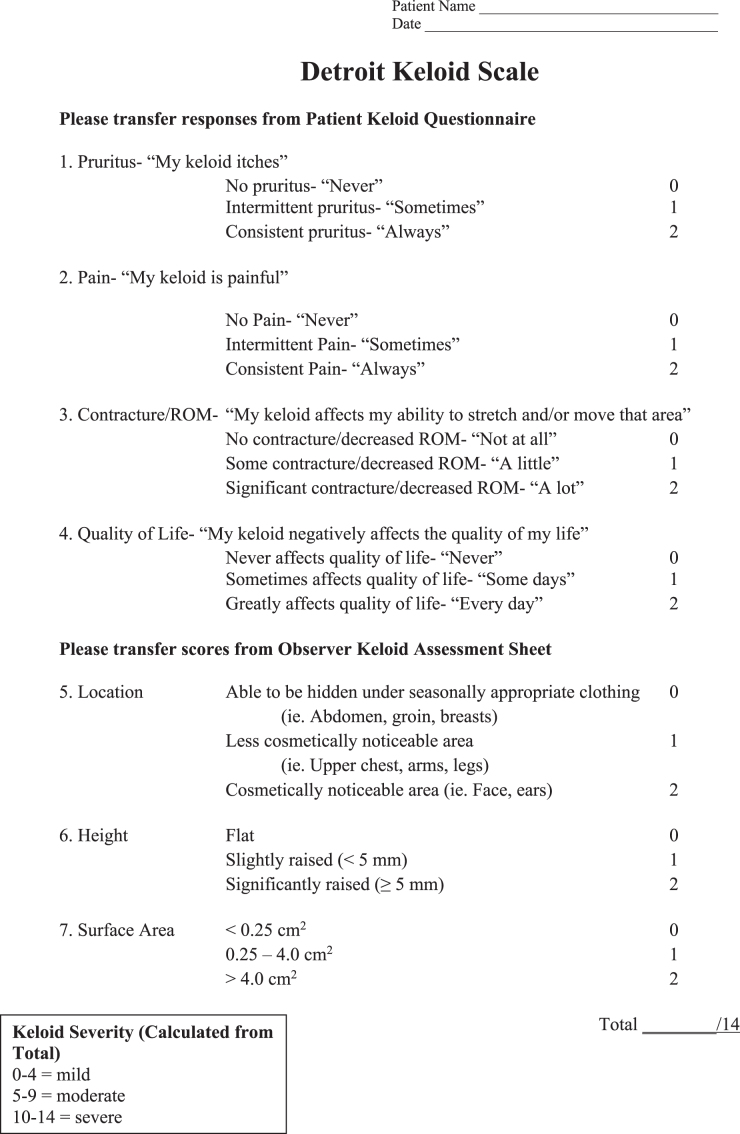
Detroit Keloid Scale.

A total of 47 physicians from the following specialties completed the survey: dermatology (25), plastic surgery (10), otolaryngology (9), primary care (1), general surgery (1), and oncology (1). The key variables reported for evaluating keloid severity included pain, area, location, contracture/range of motion, quality of life, pruritus, and height. These components were compiled into a Patient Keloid Questionnaire and Observer Keloid Assessment of the DKS (Fig. 1).

### Validation of the DKS

This study was approved by the Institutional Review Board at Henry Ford Hospital (IRB No. 11927). International Conference of Harmonization Guidelines, Declaration of Helsinki Guidelines, and Good Clinical Practice were followed in the conduct of this study. Informed consent was obtained before all study procedures. Subjects with keloid scars completed one study visit, which took place at Henry Ford Hospital, Detroit, MI at the Department of Dermatology.

Photographs were obtained of the keloid to be assessed. Patients completed the DLQI and the patient portion of the DKS and POSAS. Three physicians (two board-certified dermatologists [D.M.O. and H.W.L.] and one board-certified otolaryngologist and facial plastic surgeon [L.R.J.]) separately examined each patient's keloid in-person and completed the VSS and observer portion of the DKS and POSAS.

### Statistical analysis

Statistical analyses were performed by the department of public health at Henry Ford Hospital. Inter-rater reliability was defined as “the extent of agreement between three observers” and was assessed by computing the intraclass correlation coefficient (ICC) using a two-way mixed model with measures of consistency. An ICC within the range of 0–0.20 was considered as “slight,” 0.21–0.40 as “fair,” 0.41–0.60 as “moderate,” 0.61–0.80 as “substantial,” and 0.81–1.0 as “almost perfect.”

Pearson's correlations were used to evaluate the convergent validity where 0.9–1 indicated “very high” correlation, 0.7–0.9 indicated “high” correlation, 0.5–0.7 indicated “moderate” correlation, and below 0.5 indicated “low” correlation. Further, *p*-values of <0.05 were considered significant. All statistical analyses were performed by using SAS 9.4 (SAS Institute, Cary, NC).

## Results

**Fig. 2.**
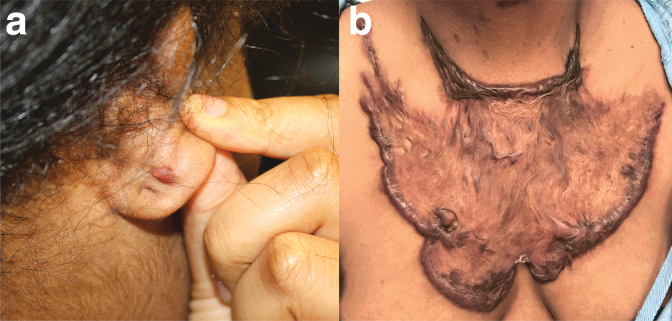
Spectrum of keloid severity in patients participating in the study: **(a)** patient with average Detroit Keloid Scale score of 2.7 and **(b)** patient with average Detroit Keloid Scale score of 11.7.

Fifty-two subjects were evaluated independently by the same three ABMS board-certified physicians experienced in caring for keloid patients (Fig. 2). The mean total score of the VSS was 6.0 ± 2.3. The mean score of the observer component of POSAS was 21.2 ± 8.2, and with additional patient component of POSAS was 60.3 ± 17.4. The mean score of the observer component of DKS was 3.3 ± 1.5, and with additional patient component of DKS was 6.0 ± 3.0. The mean total score of the DLQI was 5.0 ± 4.4.

The inter-rater reliability (Table 1) was “substantial” for observer component of the DKS and “moderate” for the VSS and observer POSAS (ICC were 0.80, 0.60, and 0.47, respectively). Pearson's correlation indicated a “moderate” association between the observer component of DKS with observer component of POSAS (*ρ* = 0.56, *p* < 0.001) and a “substantial” relationship between the observer component of DKS and VSS (*ρ* = 0.63, *p* < 0.001) (Table 2**)**.

**Table 1.** Inter-rater reliability of the Detroit Keloid Scale, Patient and Observer Scar Assessment Scale, and Vancouver Scar Scale

***Outcome measure***	***ICC (95% CI)***
DKS observer	0.801 (0.664–0.884)
DKS total	0.951 (0.904–0.973)
POSAS observer	0.466 (0.226–0.655)
POSAS total	0.877 (0.735–0.937)
VSS	0.599 (0.438–0.733)

CI, confidence interval; DKS, Detroit Keloid Scale; ICC, intraclass correlation coefficient; POSAS, Patient and Observer Scar Assessment Scale; VSS, Vancouver Scar Scale.

**Table 2.** Correlations between the Detroit Keloid Scale, Patient and Observer Scar Assessment Scale, Dermatology Life Quality Index, and Vancouver Scar Scale

	***Pearson's correlation coefficient (95% CI)***	***p***
Observer component DKS vs. observer component POSAS	0.56 (0.44–0.66)	<0.001
Observer component DKS vs. VSS	0.63 (0.52–0.72)	<0.001
Observer component POSAS vs. VSS	0.66 (0.57–0.74)	<0.001
Patient component DKS vs. patient component of POSAS	0.61 (0.50–0.70)	<0.001
Patient component DKS vs. DLQI	0.60 (0.40–0.75)	<0.001
Total DKS vs. Total POSAS	0.65 (0.54–0.73)	<0.001

DLQI, Dermatology Life Quality Index.

Pearson's correlation indicated a “moderate” association between the patient portion of DKS and patient portion of POSAS as well as the patient portion of the DKS and DLQI (0.61 and 0.60, respectively, *p* < 0.05). The observer components of the DKS and POSAS showed “moderate” correlations with the patient portions of DKS and POSAS (rater's average correlations were 0.47 and 0.50, respectively). In addition, the DKS total score consistently showed significant “substantial” relationship with POSAS total score (*ρ* = 0.65, *p* < 0.001).

## Discussion

The DKS was developed in conjunction with multidisciplinary keloid experts from around the world who treat diverse patient populations. The most important scale domains were identified, and the scale was finalized with the survey results. The DKS was successfully validated against the VSS, POSAS, and DLQI. The observer inter-rater reliability of the DKS was superior to both the VSS and POSAS.

The findings from development of the DKS are consistent with and extend from prior reports, including the Japan Scar Workshop (JSW).^11^ The JSW created the JSW Scar Scale (JSS), but in contrast to DKS, the JSS contains no patient-reported outcomes and aimed to diagnose and distinguish between keloids, hypertrophic scars, and mature scars.^11^

Other scoring systems, including the DLQI, have only patient-reported outcomes whereas some, including the VSS, have only observer components. Although the POSAS has both patient-reported and observer assessments, it is not specific for keloid scars, which are biologically distinct from hypertrophic scars, and disproportionally affect SOC patients. Thus, the DKS was created to incorporate patient-reported and observer assessments to meet the unmet need for a keloid-specific outcome measurement tool.

In addition, objective measurement tools including ultrasound have been used to measure factors such as vascularity,^4^ but these can often be costly, time consuming, and require specialized training to use in clinical practice and trials.^3^ Furthermore, vascularity was not included in DKS as it is not a prominent feature and can be difficult to assess in patients with SOC, the predominant group that develops keloids.

The observer inter-rater reliability of the DKS was superior to both the VSS and POSAS. This suggests that the DKS could be better for use in multicenter clinical trials and for those involving multiple raters and different specialties. In addition, the “almost perfect” DKS inter-rater reliability may lead to quick adoption as the standard tool for assessing keloid clinical trial outcomes.

Limitations to this study included that it was a single-center study, and there was no intra-rater reliability analysis as patients were seen at only one visit. In addition, it remains to be seen the extent that the DKS can pick up smaller changes before and after treatment in patients with more severe keloids.

In conclusion, the DKS offers a validated keloid-specific outcome measure for standardizing and comparing results. This will provide physicians, researchers, and other health care providers with a tool to better compare keloid treatment modalities. Further studies should examine DKS over time and compare results using DKS before and after treatment as well as between different treatment modalities.
